# Oligonucleotide-Peptide Conjugates: Solid-Phase Synthesis under Acidic Conditions and Use in ELISA Assays

**DOI:** 10.3390/molecules171213825

**Published:** 2012-11-22

**Authors:** Anna Aviñó, Maria José Gómara, Morteza Malakoutikhah, Isabel Haro, Ramon Eritja

**Affiliations:** 1 Institute for Advanced Chemistry of Catalonia (IQAC-CSIC), CIBER-BBN Networking Centre on Bioengineering, Biomaterials and Nanomedicine, Jordi Girona 18, E-08034 Barcelona, Spain; Email: aaagma@cid.csic.es; 2 Institute for Research in Biomedicine (IRB Barcelona), Baldiri Reixac 10, E-08028 Barcelona, Spain; 3 Unit of Synthesis & Biomedical Applications of Peptides, Institute for Advanced Chemistry of Catalonia (IQAC-CSIC), Jordi Girona 18, E-08034 Barcelona, Spain; Email: mariajose.gomara@iqac.csic.es (M.J.G.); morteza.malakoutikhah@gmail.com (M.M.); isabel.haro@iqac.csic.es (I.H.)

**Keywords:** oligonucleotide-peptide conjugates, ELISA, solid-phase synthesis, rheumatoid arthritis, epitopes, anti-citrullinated protein/peptide antibodies, ACPA, fibrin, filaggrin, citrullinated peptides

## Abstract

Here we used solid-phase methods to prepare oligonucleotides carrying fibrin/ filaggrin citrullinated peptides. Post-synthetic conjugation protocols were successfully applied for the synthesis of oligonucleotides carrying small peptides. A stepwise protocol using acid treatment for the final deprotection allowed the preparation of polypyrimidine oligonucleotides carrying longer and arginine-rich peptides. An ELISA-based test using the oligonucleotide-citrullinated peptide conjugates was developed for the detection of anti-citrullinated protein/peptide antibodies in human serum from rheumatoid arthritis patients.

## 1. Introduction

Oligonucleotide-peptide conjugates (OPCs) are chimeric molecules composed of a nucleic acid moiety covalently linked to a peptide moiety. Covalent attachment of peptides to oligonucleotides has received considerable attention because of the potential applications of these constructs in fields ranging from therapeutics to nanotechnology. For example, oligonucleotides have been attached to a number of peptides to enhance the cellular membrane permeability [1,2,3] and to study the molecular requirements for enzyme activity [4]. In addition, OPCs have been used in several sensing systems [5]. When conjugated to peptides, oligonucleotides are more resistant to nucleases [6] than when unmodified and when linked to cationic peptides, they accelerate duplex formation [7].

To date, several protocols for the chemical synthesis of OPCs have been described [8,9,10,11,12]. Conjugation of these large and functionalized molecules is a difficult task and often hindered by several side-reactions. However, these problems are mainly solved by protecting non-participating functional groups or by modifying both peptides and oligonucleotides with additional functional groups to form the desired bonds between them. The two most popular synthetic approaches are stepwise solid-phase synthesis protocols and the post-synthetic coupling of separately prepared peptide and oligonucleotide fragments.

In the stepwise solid-phase protocols, the peptide and oligonucleotide fragments are usually assembled sequentially on the same solid support. Unfortunately, the chemistries of peptide and oligonucleotide synthesis are not compatible, thus modifications of standard protecting groups or activating and deblocking agents are required [13]. In general the peptide moiety is synthesized first using *tert*-butoxycarbonyl (Boc)-amino acids with side chains protected with base-labile groups such as trifluoroacetyl (TFA) or 9-fluorenylmethoxycarbonyl (Fmoc) groups. The oligonucleotide sequence is then assembled using standard DNA synthesis protocols.

Post-synthetic coupling in solution is accomplished by chemoselective ligation mediated by mutually reactive groups that are introduced into the oligonucleotides and peptides. This strategy includes the formation of several linkages such as disulfide, amide, oxime, thiazolidine, urea and carbonyl linkages, or Diels-Alder or Hüisgen 1,3-dipolar cycloaddition reactions [12].

Despite the above methods, versatile and high yielding methods are still required for the synthesis of OPCs. Solid-phase methods for the preparation of these conjugates offer the advantage that the excess of reagents is removed easily by filtration and only one single purification step at the end of the synthesis is needed. However, these methods are not extensively used as they call for synthesizers, and the efficiency of the synthesis may be low in the case of long or difficult peptides. Recently, a method for the efficient solid phase conjugation of commercial peptides and oligonucleotides was reported [14]. Moreover, protection of the side chain of arginine with a base-labile group is complex; however, two methods have been described: the di-Fmoc derivative of arginine [15] and the introduction of ornithine followed by post-synthetic guanidylation of ornithine to arginine [16].

Peptides are used in the diagnosis of several human diseases. Synthetic peptides have been shown to be valuable tools for laboratory diagnosis and can provide uniform, chemically well-defined antigens for antibody analysis, thereby reducing inter- and intra-assay variation. The main aim in the development of peptide-based diagnostic tests is to achieve recognition of specific antibodies induced by whole viral proteins but using selected short fragments containing the most potent antigenic determinants. The success of this approach depends on the extent to which synthetic peptides mimic the immunodominant epitopes of antigens. In recent years, synthetic peptides that mimic specific epitopes of the proteins of infectious agents have been used in diagnostic systems for various human diseases [17].

Regarding rheumatoid arthritis (RA), a common autoimmune disease characterized by chronic inflammation of the synovial joints, citrullinated peptides have been successfully used for the detection of anti-citrullinated protein/peptide antibodies (ACPAs), the most specific serologic markers of this desease [18]. Citrullination is a post-transcriptional modification of proteins, in which a positively charged Arg is deiminated and converted into a neutral amino acid (citrulline) by means of peptidylarginine deiminases [19].

**Figure 1:**
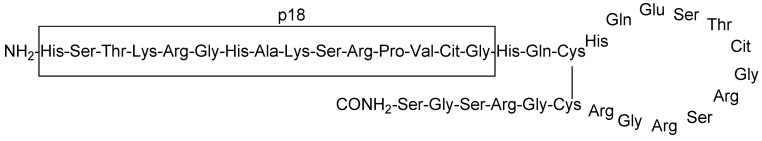
Amino acid sequence of chimeric fibrin/filaggrin citrullinated peptide (CFFCP1). Linear fibrin (p18) is indicated.

ACPAs can be detected by using enzyme-linked immunosorbent assays (ELISAs) with different citrullinated protein or peptide antigenic substrates. We have designed chimeric and cyclic peptides that bear various citrullinated peptide sequences within the same molecule [20,21]. Particularly, a chimeric fibrin/ filaggrin citrullinated peptide, [Cit630]αfibrin(617-631)-*S*306, *S*319*cyclo*[Cys306,319, Cit312]filaggrin (304-324) (CFFCP1), containing an α-fibrin peptide and the cyclic filaggrin peptide (Figure 1), which forms the basis of the commercial CCP1 test [22], is one of the most reactive ACPA epitopes [20,21].

Here we describe the synthesis of oligonucleotide conjugated to CFFCP1. In addition, the synthesis of oligonucleotides conjugated to both linear fibrin/ filaggrin [Cit630]αfibrin(617-631)-[Cys306,319, Cit312]filaggrin (304-324) and linear fibrin [Cit630]αfibrin (617-631), sequences is reported.

These conjugates are bound either directly or through complementary oligonucleotides to surfaces to design potential ELISAs to determine the presence of ACPAs in human serum. The oligonucleotide moiety may act as a reversible spacer between the surface and the antigen, thus facilitating the functionalization of the surface [23]. This strategy known as DNA-directed immobilization (DDI) [24] or oligo-tag [25] provides a chemically mild process for the binding of multiple antigens to a solid support, using DNA surfaces as immobilization matrices. This strategy has been used to immobilize proteins [26], steroids [23] and glycoconjugates [27,28,29] onto surfaces for simultaneous detection of multiple analytes.

The synthesis of these conjugates is a challenging task as the CFFCP1 peptide is relatively large (36 amino acids) and contains five arginine and two citrulline residues and a disulfide bond. The synthesis of these conjugates was attempted following post-synthetic or solid-phase protocols. Post-synthetic conjugation protocols were successfully applied to synthesize the oligonucleotide-linear fibrin peptide conjugate but were not efficient for the synthesis of the longer oligonucleotide-chimeric peptide conjugates. A new protocol for the stepwise synthesis of oligonucleotide-linear fibrin peptide conjugates using an acidic treatment for the final deprotection step is described, as the presence of five arginines in the peptide sequence precludes the use of the base-labile protecting groups [8,9,10,11,12]. In addition, the formation of the disulfide bond between the two cysteine residues of CFFCP1 was assessed after removal of the oligonucleotide-peptide conjugate from the solid support. Furthermore, we prepared several conjugates in which the two cysteines of the peptide were substituted by two serines and evaluated in an ELISA. Finally, an ELISA-based assay was used for the detection of ACPAs in RA patient sera. To this end, and to avoid unpredictable orientation on the surface, OPC immobilization on the microwell surface was achieved using oligonucleotides carrying amino or thiol groups. ACPA-OPC binding was analyzed using a well-established peroxidase-based colorimetric assay [20,21].

## 2. Results and Discussion

### 2.1. Synthesis of OPCs via Post-Synthetic Method

The synthesis of OPCs is a difficult task, especially when the peptides or oligonucleotides involved are large. The preparation of these conjugates is hindered by the fact that the protection schemes used in the synthesis of these molecules are not compatible. For example, standard protective groups in solid-phase peptide synthesis require acidic conditions for their removal, which may cause the loss of the nucleobases, especially in purines (depurination). On the other hand, oligonucleotides are usually prepared in basic conditions, which may hydrolize peptide bonds or cause unwanted side-reactions, such as racemization. The incompatibility between peptides and oligonucleotides can be circumvented by preparing the two components separately using standard peptide and oligonucleotide synthetic methods and afterwards linking them. Nevertheless, selectivity between reactive functional groups of these two components is not always accomplished, and several side reactions may occur. In addition, the secondary structure of these biomolecules may prevent the conjugation reaction.

The peptides carrying a maleimido group used in this study were synthesized following the Fmoc/ *tert*-butyl (*t*-Bu) strategy [30]. CFFCP1 peptide was also prepared in linear form, the two cysteines being protected with an acetamido group. Once the assembly of the peptide sequences was completed, cleavage and deprotection of the resulting peptide supports was carried out using the usual acid treatment (TFA). The peptides were isolated by precipitation, purified by analytical HPLC, and characterized by electrospray mass spectrometry. Disulfide bond formation was accomplished by removing acetamido groups in the presence of iodine in acidic conditions. The resulting cyclic CFFCP1 peptide carrying the maleimido group was isolated by HPLC and characterized by mass spectrometry.

Two oligonucleotides containing a hexyl thiol group at the 5′-end were prepared. The first synthesis was an octathymidine oligonucleotide and the second the 20-mer sequence (5′-TACATGCGTGCTGATGCAAG-3′).

**Figure 2:**
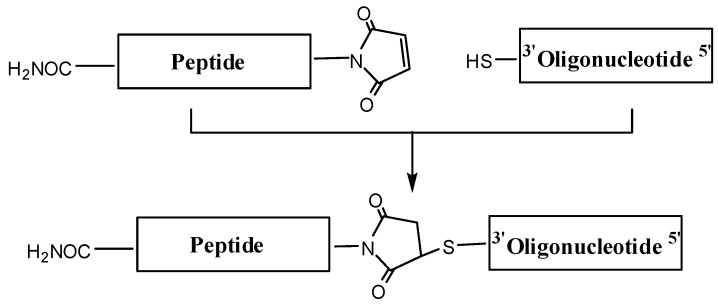
Synthesis of OPCs using maleimido-peptides and thiol-oligonucleotides following the post-synthetic conjugation protocol.

Maleimido-peptides were reacted with thiolated oligonucleotides (Figure 2), and the expected conjugates were isolated by HPLC and characterized by mass spectrometry. Moderate yields of the short OPC were obtained following this strategy, but conjugation of the 20mer sequence with cyclic peptide CFFCP1 was unsuccessful. Maleimido–thiol linkage is commonly used to connect short peptides to oligonucleotides; however, some problems have been reported for complex peptides. For example, a decrease in efficiency has been described with hydrophobic peptides that are not highly soluble in aqueous solvents [11]. Also, low yields have been observed during the conjugation of oligonucleotides to highly structured peptides with several positive charges [11]. In our case, during the HPLC purification of the reaction between the maleimido-CFFCP1 cyclic peptide and thiol-oligonucleotides, we observed conversion of the OPC to the starting thiol-oligonucleotide and maleimido-peptide by a retro-Michael reaction. This reaction was also detected in a conjugate of an oligonucleotide with a long peptide [31], but the degradation of the conjugates was not as rapid as observed during the conjugation to CFFCP1. To obtain the desired conjugates, we followed the solid-phase strategy.

### 2.2. Synthesis of Linear OPCs via Solid-Phase Method

Most of the solid-phase methods reported for OPC synthesis contain the peptide moiety at the 3′-end of the oligonucleotide [32,33,34]. In these strategies, the peptide is first synthesized and then the oligonucleotide is assembled in the peptidyl resin. Two types of *N*-protected amino acids can be used. First, the *N*-α-Boc-amino acids containing base-labile protecting groups (Fmoc, Fm, Tfa, Tos, Dnp) for the protection of trifunctional amino acid side chains. These groups can be removed concomitantly with the nucleotide protecting groups after the completion of OPC synthesis [32,33]. The second strategy uses Fmoc-amino acids containing acid-labile (*t*-Bu) groups for the protection of trifunctional amino acid side chains. The *t*-Bu groups are removed without the presence of the oligonucleotide. Several authors have also proposed various amino acid side chain protecting groups that can be removed in mild conditions [12]. As CFFCP1 peptide has five arginines, which have no satisfactory side chain protecting group that can be removed in basic conditions, we tested a new protocol for the stepwise synthesis of OPCs using an acid treatment for the final deprotection step. In this way, the standard Fmo/*t*-Bu strategy was used to synthesize the peptide; however, this method requires the use of TFA in the presence of the oligonucleotide, which may induce depurination. Although some authors have described the isolation of OPCs after TFA treatment [34], we found that purine 2′-deoxynucleotides are not stable to TFA [35]. For this reason the method may be restricted to polypyrimidine sequences [35,36].

**Figure 3:**
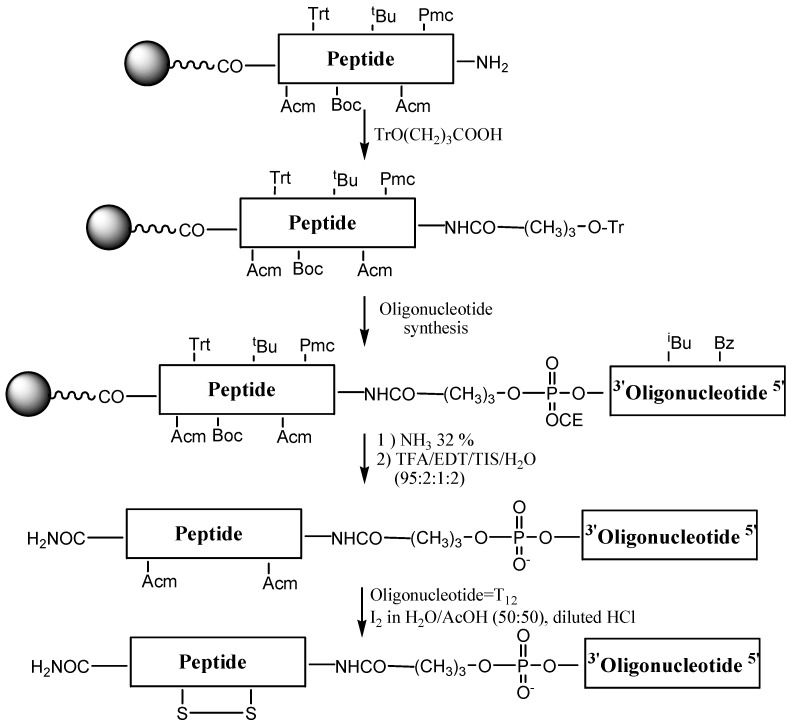
Synthesis of OPCs using the stepwise solid-phase method.

Syntheses of OPCs carrying p18 and CFFCP1 sequences were carried out following the Fmoc/*t*-Bu strategy (Figure 3). First, the peptide with acid-labile side chain protection was assembled using the following groups for the protection of the side chains of amino acids: triphenylmethyl (Trt) for histidine; *tert*-butyl (*t*-Bu) for serine and threonine; 2,2,5,7,8-pentamethyl-chroman-6-sulfonyl (Pmc) for arginine, *tert*-butoxycarbonyl (Boc) for lysine and acetamidomethyl (Acm) for cysteine. Then 4-*O*-trityl-4-hydroxybutyric acid linker was added to the *N*-terminal position of the peptide support [10,13]. This linker generates a free hydroxyl after the removal of the trityl group, thus allowing assembly of the oligonucleotide sequence on a DNA synthesizer. The removal of the trityl group is done at the DNA synthesizer using the standard detritylation reagent (2% TCA in dichloromethane). The resulting oligonucleotide-peptide support contains protecting groups that are removed with bases (nucleobases and phosphate groups) and with acids (amino acid side chain protecting groups and removal of the OPC from the solid support). The order of these treatments was assayed on a model sequence. Octathymidinyl-CFFCP1 linear OPC (OPC-4) was obtained following both protocols. First, we used ammonia followed by TFA treatment in the presence of scavengers. The second protocol consisted of the deprotection of the OPC with TFA, followed by ammonia treatment. The Acm groups are stable to oligonucleotide and peptide synthesis conditions obtaining the OPC carrying Cys(Acm) residues. The conjugates were purified by HPLC and analyzed by mass spectrometry (Table 1). Both protocols gave the desired conjugate as a major product but the yield was higher in the first. Nevertheless, this method allows the synthesis of only pyrimidine sequences, as these are stable to TFA [35,36].

**Table 1:** Oligonucleotide peptide conjugates prepared in this study.

Name	Method	Sequence ^C^Peptide^N^-link-(5′-3′)	MS (expected)	MS (found)
OPC-1	PSC	p18-X-TTTTTTTT	4438	4435
OPC-2	PSC	p18-X-TACATGCGTGCTGATGCAAG	8211	8208
OPC-3	PSC	(CFFCP1)-X-TACATGCGTGCTGATGCAAG	10673	10677
OPC-4	SSPS	(CFFCP1 linear)-Y-TTTTTTTT	6687	6688
OPC-5	SSPS	(CFFCP1 linear)-Y-TCTCTCTCTCTC	7814	7820
OPC-6	SSPS	(CFFCP1 linear)-Y-TTTTTTTTTTTT	7904	7910
OPC-7	SSPS	(CFFCP1)-Y-TTTTTTTTTTTT	7759	7765
OPC-8	SSPS	(CFFCP1Ser )-Y-TTCTTTTCTCTT	7684	7686
OPC-9	SSPS	(CFFCP1Ser )-Y-***UUCUUUUCUCUU***	7918	7914

***C***, ***U*** are 2′-*O*-methyl-RNA nucleotides; X is the maleimido-thiol linkage; Y is the 4-hydroxybutanoic linker; PSC stands for post-synthetic conjugation; SSPS stands for stepwise solid-phase synthesis; p18 is ^N^His-Ser-Thr-Lys-Arg-Gly-His-Ala-Lys-Ser-Arg-Pro-Val-Cit-Gly^C^-CONH_2_; CFFCP1 is shown in Figure 1. CFFCP1 linear is ^N^His-Ser-Thr-Lys-Arg-Gly-His-Ala-Lys-Ser-Arg-Pro-Val-Cit-Gly-His-Gln-Cys(Acm)-His-Gln-Glu-Ser-Thr-Cit-Gly-Arg-Ser-Arg-Gly-Arg-Cys(Acm)-Gly-Arg-Ser-Gly-Ser^C^-CONH_2_; CFFCP1Ser is ^N^His-Ser-Thr-Lys-Arg-Gly-His-Ala-Lys-Ser-Arg-Pro-Val-Cit-Gly-His-Gln-Ser-His-Gln-Glu-Ser-Thr-Cit-Gly-Arg-Ser-Arg-Gly-Arg-Ser-Gly-Arg-Ser-Gly-Ser^C^-CONH_2_.

**Figure 4:**
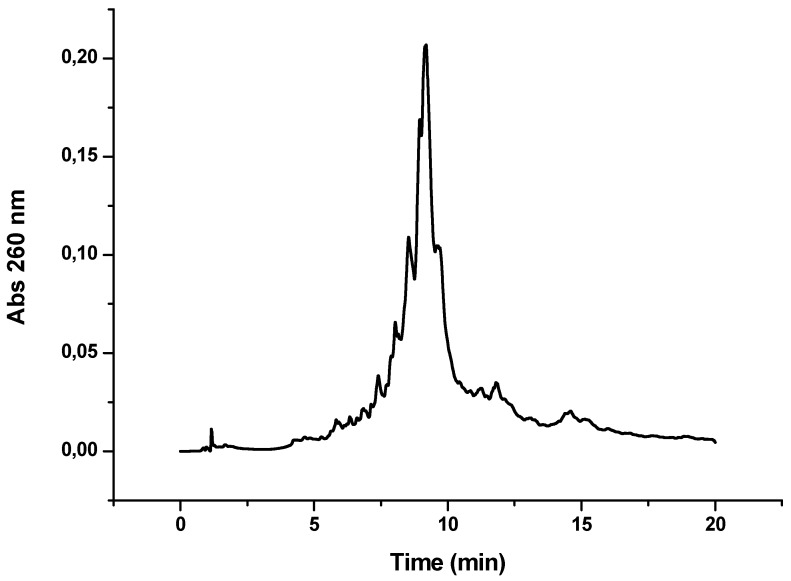
HPLC profile obtained after the synthesis of OPC-8 (CFFCP1Ser)-Y-TTCTTTTCTCTT). Detection wavelength 260 nm.

OPC-8 and -9 were prepared following a solid-phase method. As an example, in Figure 4 is shown the HPLC profile of OPC-8. A new peptide (CFFCP1Ser) was synthesized in which the two Cys are substituted for Ser. This linear peptide proved also to recognize ACPAs in human serum from RA patients (data not shown), according to results published by Sebbag *et al.* [37], who described that the citrullinated derived peptide of the 306-324 C-terminus of filaggrin with Ser in positions 306 and 319 was recognized by a high number of RA sera.

After the addition of the 4-hydroxybutanoic linker, two sequences were assembled in CFFCP1Ser peptidyl solid support. The first was a DNA sequence containing dC and T derivatives (OPC-8) and the second is an RNA containing 2′-*O*-methyl-C and 2′-*O*-methyl-U derivatives with the same sequence (OPC-9). These two conjugates were deprotected using ammonia followed by the TFA protocol and used for the immunological test of RA.

### 2.3. Synthesis of Cyclic Peptide Oligonucleotide Conjugate Using the Solid-Phase Method

The strategy of peptide cyclization has been described for designing citrullinated peptides with higher affinity for the ACPA. The cystine-bridged cyclic peptides led to far better results in RA diagnosis than the linear counterparts [22].

**Figure 5:**
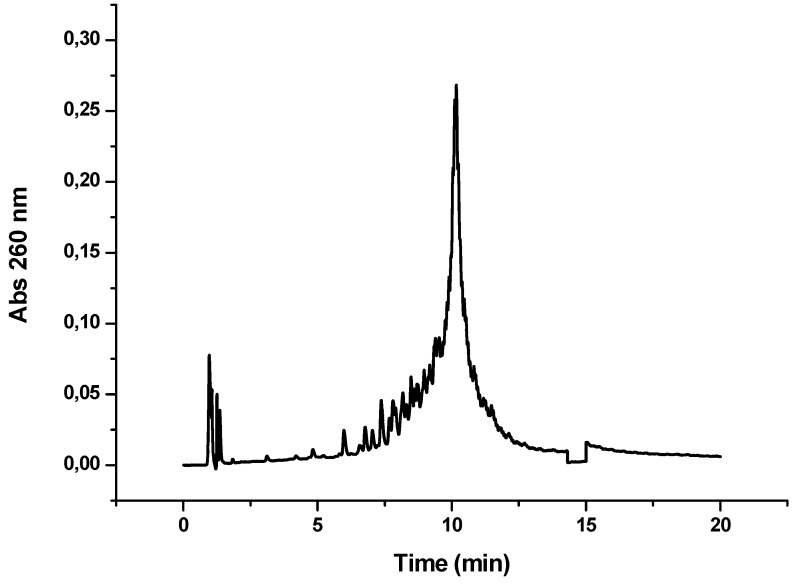
HPLC profile obtained after the synthesis of OPC-7 (CFFCP1-Y-T_12_). Detection wavelength 260 nm.

OPC-7 corresponds to a cyclic conjugate of OPC-6. To our knowledge, this is the first time that the synthesis of a cyclic peptide has been attempted while covalently attached to an oligonucleotide. The formation of the disulfide bond was carried out as described for the cyclization of linear CFFCP1-peptide [20]. Specifically, Acm protecting groups of the Cys of OPC-6 were removed in the presence of I_2_ in acidic conditions and quenched by ascorbic acid. Finally, cyclic OPC was purified by HPLC (Figure 5) and characterized by mass spectrometry. The overall yield of cyclic OPC synthesis was low, nevertheless we were able to isolate the correct cyclic OPC for the T_12_ sequence.

### 2.4. Enzyme-linked immunosorbent assays of OPCs

**Figure 6:**
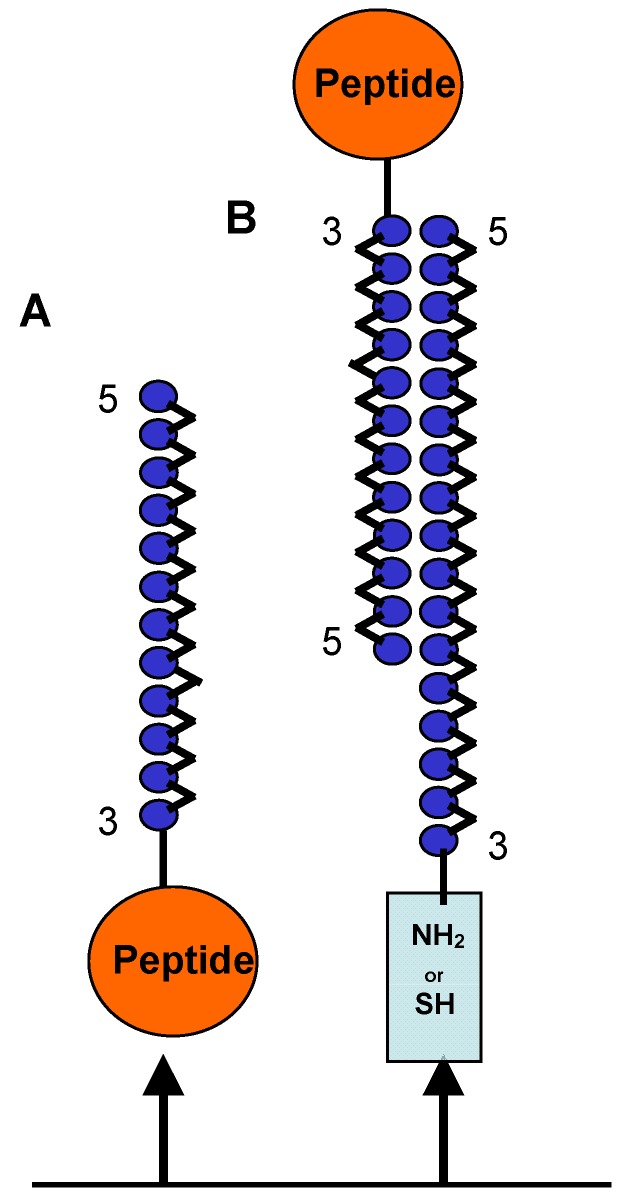
Schematic representation of two forms of OPC binding. A) Single OPC attached to ELISA plate and B) duplex OPC formed by OPCs and their complementary strand, which is attached to the ELISA plate through a thiol or an amino group.

In addition to citrullinated proteins such as filaggrin, fibrin and vimentin, several synthetic citrullinated peptides have been used as antigenic substrates in serological tests for the diagnosis of RA [17]. We considered the linear peptide CFFCP1Ser and we have prepared and evaluated OPC-8 and OPC-9 in ELISAs for the detection of ACPAs present in the human serum of RA patients. We analyzed two ELISA formats. The first one was based on the binding of a single OPC to the ELISA plate (Figure 6A). While the second was based on a duplex format in which a single OPC was hybridized with its complementary DNA strand and the duplex was attached to the ELISA plate (Figure 6B).

OPC-8 and OPC-9 gave the same results in both ELISA formats using a peroxidase-based colorimetric read-out. Immunological tests were carried out at two concentrations (25 pM and 250 pM per well) of single and duplex OPC and two different binding chemistries were used to attach them to the surface of ELISA plate. In the first approach we used DNA-Bind^TM^ plates (Costar, Corning Inc., Corning, NY, USA) coated with reactive *N*-oxysuccinimide esters, which react with nucleophiles such as amines in basic conditions to form amide groups. The single OPCs reacted through free amino groups of the peptide sequence whereas in duplex OPCs, in addition to these amino groups the complementary DNA strand of OPC contained a free amino in the 5′-end.

**Figure 7:**
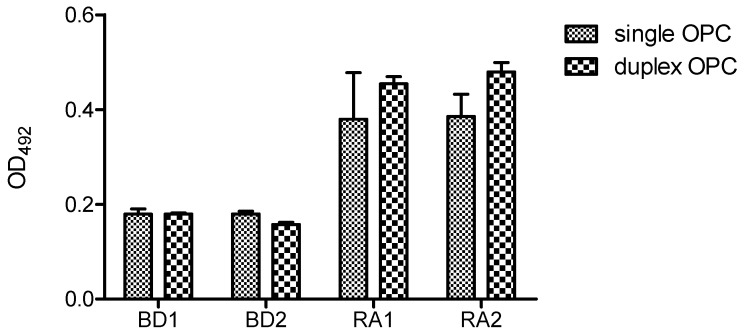
Reactivity of rheumatoid arthritis (RA) and blood donor (BD) sera with single and duplex OPC (OPC-8 with its complementary strand 5′AAGAGAAAAGAATTTTT-NH_2_ 3′). A concentration of 25 pmol/well of each OPC was immobilized via amine groups on DNA-Bind^TM^ plates.

Figure 7 shows the ELISA results of single and duplex OPCs with human serum at 25 pmol/well using DNA-Bind^TM^ plate. Two serum samples from healthy volunteers (BD1 and BD2) and two from ACPA-positive (RA1, RA2) RA patients were analyzed. In these conditions both formats (single OPC and duplex OPC) were recognized by ACPAs, although low optical density values were obtained. The reactivity of the two RA sera was slightly lower for single format than the duplex OPC. Moreover, single OPCs showed higher variability of the intra-assay replicated values.

**Figure 8:**
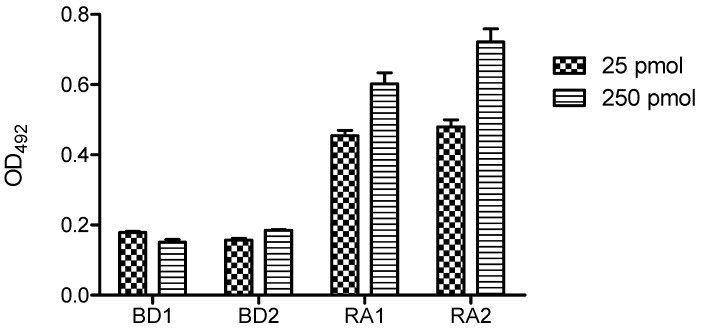
Reactivity of rheumatoid arthritis (RA) and blood donor (BD) sera with duplex OPC (OPC-8 with its complementary strand 5′AAGAGAAAAGAATTTTT-NH_2_ 3′). Concentration of 25 and 250 pmol/well of duplex OPC-8 was immobilized via amine groups on DNA-Bind^TM^ plates.

Since the concentration of OPC tested (25 pmol/well) was about 10-fold lower than the peptide concentration used in the previously reported ELISAs [10,21], next we tested 250 pmol/well of duplex OPC following the same experimental procedure. The reactivity of RA sera was now significantly higher, while BD control sera remained low (Figure 8). In these conditions, the differences between ACPA-positive and control sera increased significantly although the optical density values were lower than those obtained with the peptide-based assay.

With the aim of improving these results, the ELISA procedure assay was modified in order to properly present the antigenic sequence of the conjugate for its specific ACPA recognition. To this end, an oriented covalent bonding of the duplex OPC to the solid surface by means of a thiol group at the 5′-end of its complementary strand was assayed. The immobilization of a concentration of 250 pmol/well of this duplex was performed in neutral conditions using an ELISA plate Nunc Immobilizer^TM^ (Thermo Scientific, Roskilde, Denmark). These commercial ELISA plates contain a stable electrophilic group that reacts with nucleophiles such as free amines, thiols or hydroxy-groups. Reactivity of the amine and thiol groups is a function of pKa and can be modulated by pH. In neutral conditions, amines are protonated and are poor nucleophiles because the pH is below their pKa. Nevertheless, in neutral conditions thiolate anion is also formed and should be the reactive species. Thus, the activated plate should react only with the thiol group at the 5’-end of the complementary strand of the duplex OPC.

**Figure 9:**
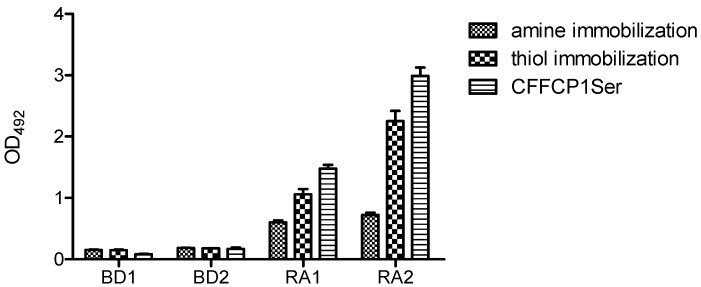
Reactivity of rheumatoid arthritis (RA) and blood donor (BD) sera with duplex OPC. For amine immobilization OPC-8 with its complementary strand 5′AAGAGAAAAGAATTTTT-NH_2_ 3′ was used while for thiol immobilization OPC-8 with 5′ AAGAGAAAAGAATTTTT-SH 3′ was used. The peptide CFFCP1Ser was added as positive control. A concentration of 250 pmol/well of duplex OPC-8 was immobilized on the corresponding ELISA plates.

Figure 9 shows the comparative results obtained with the amine and thiol immobilization of the duplex OPC on the solid surface. Thiol immobilization was more effective than amino immobilization. Nevertheless, OPC with a complementary amino strand also gave a positive signal. Although OPC immobilization occurs mainly throughout covalent bonding to the activated plate, unspecific non-covalent binding should also be assumed. It should be highlighted that the oriented presentation of the antigenic sequence of duplex OPC by means of a defined chemical strategy led to a significant increase in specific ACPA recognition.

These results demonstrated that the immune recognition of ACPAs present in the human serum of RA patients is also possible using OPCs. Optimized conditions were found at 250 pmol and thiol immobilization of the duplex OPC.

Furthermore, we compared the reactivity of the two RA serum samples with the peptide substrate (CFFCP1Ser) using the optimized conditions. As illustrated, the recognition of peptide CFFCP1Ser was 20–25% higher than that corresponding to OPC8. This enhancement may be attributable to a lower binding efficiency of OPC to the ELISA plate or to a lower stability of the oligonucleotide-peptide conjugate in the ELISA assay. Nevertheless, here we have demonstrated the capacity of OPCs to recognize ACPAs present in human serum of RA patients. These antigenic conjugates might be of relevance in the development of new nanotechnology-based immunosensors for the specific detection of biomarkers with applications in the diagnosis of human diseases.

## 3. Experimental

### 3.1. Synthesis of the MBA-p18 and MBA-CFFCP1 Peptide

The [Cit630]αfib(617-631) peptide (p18) and [Cit630]αfibrin(617-631)-[Cys306,319, Cit312]filaggrin(304-324) peptide (CFFCP1) were synthesized manually as C-terminal carboxamides on a Tentagel RAM resin (Rapp Polymere GmbH, Tübingen, Germany) (500 mg, 0.28 meq/g) and following a 9-fluorenyl-methoxycarbonyl (Fmoc) strategy. Amino acid side chain protection was effected by the following: triphenylmethyl (Trt) for histidine; *tert*-butyl (*t*-Bu) for serine and threonine; 2,2,5,7,8-pentamethyl-chroman-6-sulfonyl (Pmc) for arginine, *tert*-butoxycarbonyl (Boc) for lysine and acetamidomethyl (Acm) for cysteine.

Sequences were: MBA-p18, maleimido-benzoyl-His-Ser-Thr-Lys-Arg-Gly-His-Ala-Lys-Ser-Arg-Pro-Val-Cit-Gly-CONH_2_ and MBA-CFFCP1, maleimido-benzoyl-His-Ser-Thr-Lys-Arg-Gly-His-Ala-Lys-Ser-Arg-Pro-Val-Cit-Gly-His-Gln-Cys(Acm)-His-Gln-Glu-Ser-Thr-Cit-Gly-Arg-Ser-Arg-Gly-Arg-Cys(Acm)-Gly-Arg-Ser-Gly-Ser-CONH_2_.

The coupling reaction was performed using four-fold molar excesses of activated Fmoc-amino acids throughout the synthesis. The amino acids were activated essentially by means of treatment with 2-(1*H*-7-azabenzotriazole-1-yl)-1,1,3,3-tetramethyl uronium hexafluorophosphate methanaminium (HATU) and a base such as diisopropylethylamine (DIPEA). The Fmoc-deprotection step was done twice with 20% piperidine in dimethylformamide (DMF) for 10 min. The efficiency of these reactions was evaluated by the ninhydrine colorimetric reaction.

The peptide sequence was subsequently derivatized in *N*-terminus with 3-(2,5-dioxo-2,5-dihydro-pirrol-1-yl)-benzoic acid. The *m*-maleimidobenzoic acid was incorporated in solid phase by means of treatment with *N*,*N*′-diisopropylcarbodiimide (DIPCDI) and hydroxybenzotriazole (HOBt). The coupling reaction was carried out in duplicate using a three-fold excess of reagents.

Once the synthesis was completed, the cleavage and deprotection processes of the peptidyl resin were carried out by means of treatment with 94% trifluoroacetic acid (TFA) for 4 h in the presence of 2.5% H_2_O, 2.5% 1,2-ethanedithiol (EDT) and 1% triisopropylsilane (TIS) as scavengers.

CFFCP1 peptide was synthesized in its linear form, in which the Cys are protected by Acm groups, and in cyclic form, in which the two Cys form a disulfide bond.

The peptides were isolated by precipitation with cold diethyl ether, centrifuged and lyophilized. They were then characterized by analytical HPLC on a Kromasil C-18 column (Teknokroma, San Cugat del Vallés, Spain, 5 μm, 25 × 0.46 cm) with a linear gradient of 95–5% solvent A in solvent B over 20 min at a flow rate of 1 mL/min using 0.05% TFA in water (A) and 0.05% TFA in acetonitrile (ACN) (B) as eluting system. The peptide was purified by preparative HPLC in a Kromasil-C8 column (Tecknokroma, 5 μm, 25 × 2.2 cm) and characterized by Electrospray-Mass Spectrometry (ES-MS, positive mode) MBA-p18: [M] = 1872.68 (expected M = 1873.08). MBA-CFFCP1 linear [M] = 4334.76 (expected M = 4333.20). Electrospray ionization mass spectrometry was performed with a Liquid Chromatograph-Time of Flight (LC-TOF) detector, LCT Premier XE (Micromass Waters, Manchester, UK) coupled to the UPLC (Waters, Milford, MA, USA). Samples were dissolved in a mixture of acetonitrile/water (1:1, v/v) and analyzed previously at the UPLC with a flow rate of 0.3 mL/min. Mass spectra were recorded in positive ion mode in the *m/z* 500–2500 range.

### 3.2. Synthesis of Oligonucleotides

Oligonucleotides 5′ TTTTTTTT 3′ and 5′ TACATGCGTGCTGATGCAAG 3′ were synthesized in a 0.2 µM scale using DNA synthesizer (Applied Biosystems 3400, Foster City, CA, USA). After assembly of the sequences, a thiol group was introduced at the 5′-end using thiol modifier C6 S-S-CE phosphoramidite (Link Technologies, Lanakshire, Scotland). The oligonucleotides were deprotected using 1 mL of 33% NH_3_ and 7 mg of 1,4-dithiothreitol (DTT) for 6 h at 55 °C. After, the solution was concentrated desalted on a Sephadex column (NAP-10) prior to use.

Complementary oligonucleotide of OPC-8 and -9 with a spacer of five thymidines was prepared following the same procedure. These sequences were prepared using 3′-Thiol-modifier C3 S-S-CPG (Link Technologies) or 3′-Amino-modifier-C7 CPG (Link Technologies) to obtain an amino or thiol group at the 3′-end. These sequences were 5′AAGAGAAAAGAATTTTT-NH_2_ 3′ and 5′AAGAGAAAAGAATTTTT-SH 3′.

### 3.3. Synthesis of Linear OPCs Using the Post-Synthetic Method

The MBA-p18 peptide carrying a maleimido group (0.3 mg) was incubated with 5′-SH-hexyl-phosphate-TTTTTTTT-3′ (2.4 OD units at 260 nm) in 0.1 M triethylammonium acetate (TEAA) at pH 7.0 overnight at room temperature. The solution was evaporated to dryness and the mixture analysed by HPLC. Column: Nucleosil 120-10 C_18_ (250 × 4 mm); 20 min linear gradient from 0% to 50% B; flow rate 3 mL/min; solution A was 5% ACN in 0.1 M aqueous TEAA and B 70% ACN in 0.1 M aqueous TEAA. The starting thiol-oligonucleotide (elution time 9 min) was converted to a new product (elution time 10.9) which was collected. For the synthesis of OPC-3, 100 µL of DMF was added to the coupling reaction to increase the solubility of the peptide. Column: X-Bridge^TM^ OST C18 (4.6 × 50 mm, 2.5 µm); 20 min linear gradient from 0% to 20% B followed by an 8 min gradient to 100% B and flow rate 1 mL/min; solution A was 5% ACN in 0.1 M aqueous TEAA and B 70% ACN in 0.1 M aqueous TEAA. Yields after HPLC purification were 5–10%. Mass spectrometry confirmed the expected mass for OPCs. Mass spectrometry (MALDI-TOF, negative mode) analysis: OPC-1 [M] = 4435 (expected M = 4438). OPC-2 [M] = 8208 (expected M= 8211). OPC-3 [M] = 10677 (expected M = 10673). OPC-3 was not stable and decomposed readily to starting thiol-oligonucleotide by a retro-Michael reaction. Matrix-assisted laser desorption ionization time-of-flight (MALDI-TOF) mass spectra were recorded either on a Voyager-DE-RP spectrometer (Applied Biosystems) or in a 4800 Plus MALDI TOF/TOF (ABSCiex-2010, Flamingham, MA, USA) in negative mode by using 2,4,6-trihidroxyacetophenone hydrate (THAP) matrix with ammonium citrate, 50 mg/mL in water as additive).

### 3.4. Synthesis of Linear OPCs Using the Solid-Phase Method

The CFFCP1 peptide and the same peptide in which the two Cys were replaced by Ser (CFFCP1Ser) were prepared in solid-phase as described before. Before adding the spacer, we analyzed a small amount of the peptide by mass spectrometry (electrospray, positive mode). CFFCP1Ser: [M] = 3967.05 (expected M = 3966.28), CFFCP1 linear: [M] = 4141.20 (expected M= 4141.57). Then, 4-hydroxybutyric acid linker was added to the *N*-terminal position of the peptide sequence using PyBOP, (5-fold excess) and DIEA (10-fold excess) for 1 h. The unreacted amino groups of the resulting support were blocked with acetic anhydride and DIEA. Next, solid-phase oligonucleotide synthesis was carried out using this peptide solid support on a DNA synthesizer (Applied Biosystems 3400). OPCs were prepared using 2-cyanoethyl phosphoramidites of thymidine and 2′-deoxycytidine for the OPCs 4,5,6,7,8 and 2-cyanoethyl phosphoramidites of 2′-*O*-methyluridine and 2′-*O*-methylcytidine for OPC-9. The following solutions were used: 0.4 M 1*H*-tetrazole in ACN (activation); 3% trichloroacetic acid in DCM (detritylation), acetic anhydride/pyridine/tetrahydrofuran (1:1:8) (capping A), 10% *N*-methylimidazole in tetrahydrofuran (capping B), 0.02 M iodine in tetrahydrofuran/pyridine/water (7:2:1) (oxidation).

A modified 1 µmol synthesis cycle was used: coupling time was increased to 5 min, capping and oxidation to 1 min, and detritylation to 2 min (4 × 30 s). The average coupling yield was around 97–98% per step for RNA monomers and 99% for DNA monomers. The solid supports were treated with 2 mL concentrated aqueous ammonia (three treatments of 30 min each). After that, acidic treatment with 95% trifluoroacetic acid (TFA) in the presence of scavengers 2% H_2_O, 2% 1,2-ethanedithiol (EDT) and 1% triisopropylsilane (TIS) for 4 h. The OPCs were isolated by precipitation with cold diethyl ether, centrifuged and lyophilized. Finally, the conjugates were desalted in a Sephadex column (NAP-10) and purified by HPLC as described above. Yields after HPLC purification were 10–20%.

OPC-4 was also obtained using first a TFA treatment and then the residue was treated with ammonia. Yields of OPC-4 using ammonia + TFA treatment were twice those achieved with TFA + ammonia treatment. The purified products were analyzed by MALDI-TOF (negative mode) mass spectrometry (see Table 1 and supplementary material). OPC-4 [M] = 6688 (expected M = 6687); OPC-5 [M] = 7820 (expected M = 7814), cal 7814; OPC-6 [M] = 7910 (expected M = 7904); OPC-8 [M] = 7686 (expected M= 7684); OPC-9 [M] = 7914 (expected M = 7918).

### 3.5. Synthesis of Cyclic OPC Using Solid-Phase Method

T_12_-CFFCP1 (OPC-7) was prepared as described for linear OPC. Formation of the disulfide bond between the two Cys was carried out selectively after the deprotection of Acm protecting group of these two Cys. Linear T_12_-CFFCP1 (0.1 mg) was dissolved in 2 mL of H_2_O:AcOH (1:1) under N_2_ atmosphere, then 600 µL HCl (1 M, 0.1 mL/mg) followed by 300 µL of I_2_ (0.1 M, 20 eq/Acm) were added. After four hours, the iodine was quenched by adding 1 M ascorbic acid drop-wise until the mixture become colorless. The solution was then concentrated by evaporation under reduced-pressure to approximately one third of the original volume. Finally, the cyclic conjugate was isolated and purified by HPLC. Yield after HPLC purification was 2%. MALDI-TOF mass spectrometry OPC-7 [M] = 7765 (expected M = 7759).

### 3.6. Serum Specimens

Human serum was collected from patients attending the Rheumatology Service at the Hospital Clinic in Barcelona. These patients were diagnosed with RA following the revised criteria formulated by the American College of Rheumatology and were previously tested for the presence of anti-CCP2 antibodies by ELISA (Immunoscan RA; Eurodiagnostica, distributed by Diasorin, Madrid, Spain). Serum samples used as negative controls were obtained from healthy blood donors at the same hospital.

### 3.7. ELISA Assays

Immunological assays were performed using ELISA microplates. Single OPCs and duplexes formed by annealing with the corresponding complementary sequence containing an amino or a thiol group in the 5’′end were used. In addition, peptide was also included to compare with OPCs in the same ELISA assay. Thiol or amino groups of the OPCs were used for attachment to the ELISA microplate. For amino groups in basic conditions, ELISA microplates (Costar Corp, DNA-bind^TM^ *N*-oxysuccinimide surface) were used as described [20]. For thiol groups microplates from Thermo Scientific Nunc Immobilizer^TM^ Amino Surface were used.

Immunological assays were performed at 25 and 250 pmol/well. In DNA-bind^TM^ microplates, peptide, duplex and OPC were dissolved in 50 mM Na_2_HPO_4_ 1 mM EDTA (pH 8.5) binding buffer whereas in Nunc microplates, peptide, duplex OPC and single OPC were dissolved in 10 mM phosphate buffer with 150 mM NaCl (pH 7) binding buffer. The rest of the protocol was the same for both ELISAs. Briefly, 100 µL of the solutions was added to each microplate wells and incubated overnight at 4 °C. In order to estimate the background reading, each plate contained control wells that included all reagents except the serum sample, while to evaluate non-specific reactions of human serum, control wells included all reagents except peptide, duplex or OPC. For blank controls, wells were coupled with 2 µg BSA/well. After incubation, the plates were blocked with 2% BSA in binding buffer for 1 h at room temperature. Human serum was diluted 50-fold in RIA buffer (1% BSA, 350 mM NaCl, 10 mMTris HCl (pH 7.6) 1% vol/vol Triton X-100, 0.5 wt %/vol Na-deoxycholate, 0.1% SDS) supplemented with 10% fetal bovine serum. 100 µL/well was added and incubated for 1.5 h at room temperature. After washing 6 times with PBS/0.05% Tween-20, 100 µL/well of antihuman IgG conjugated to peroxidase diluted 1:1,000 in RIA buffer was added. After incubation for 1 h at room temperature, the plate was washed six times with PBS/0.05% Tween-20, and bound antibodies were detected with *o*-phenylenediamine (OPD, Sigma Aldrich, St Louis, MO, USA). The plates were incubated at room temperature for 30 min. The reaction was stopped with 50 µL of 2 N H_2_SO_4_ per well, and absorbance values were measured at a wavelength at 492 nm. All human serum samples were tested in duplicate. Control sera were also included to monitor inter-and intra-assay variation.

## 4. Conclusions

Biomolecule conjugation has allowed the preparation of novel chimeric molecules with properties than cannot be achieved from single biomolecules. One of the areas of interest is the use of oligonucleotides for the binding of multiple antigens to solid supports; using DNA surfaces as immobilization matrices. The DNA-directed immobilization method has been successfully used for the patterning of surfaces with specific antibodies [38] but no data is available for the DNA-directed immobilization of peptide antigens. In order to achieve this goal, the development of methodology for the synthesis of oligonucleotide-peptide conjugates is needed. Peptides and oligonucleotides are rich in functional groups and the coupling between them is challenging. Here we described the synthesis of OPCs. Conjugation was achieved using a large and complex peptide that was previously designed and tested for RA diagnosis. We show that post-synthetic and solid-phase methods are suitable approaches to prepare these OPCs. Solid-phase synthesis was more adequate because it is more straightforward, and the yield was slightly better. Nevertheless, the strong acid treatment that is required makes this method suitable only for DNA or RNA pyrimidine sequences. Given that RNA is more stable to acids than DNA, it remains to be seen whether acid treatments can also be used for RNA sequences with purine nucleosides.

Here we described the preparation of an OPC carrying a disulfide bond in a polythymidine sequence. Cyclization was accomplished using a similar procedure as the one described for peptides after solid-phase synthesis of the corresponding linear OPC.

Finally, OPCs prepared were tested in ELISAs for RA diagnosis. Two formats, single OPCs and duplex OPCs were similarly recognized by RA patient sera containing ACPAs. Specific immobilization of duplex OPCs on the ELISA plate was more efficient using thiol than amino groups. The findings presented here can be extended to the preparation of conjugates of triplex-forming oligonucleotides as these usually comprised polypyrimidine sequences.

## Supplementary Materials

Supplementary materials can be accessed at: http://www.mdpi.com/1420-3049/17/12/13825/s1.
